# A Bird’s-Eye View of Chromosomic Evolution in the Class Aves

**DOI:** 10.3390/cells13040310

**Published:** 2024-02-07

**Authors:** Rebecca E. O’Connor, Rafael Kretschmer, Michael N. Romanov, Darren K. Griffin

**Affiliations:** 1 School of Biosciences, University of Kent, Canterbury CT2 7NJ, UK; rebeckyoc@gmail.com (R.E.O.); m.romanov@kent.ac.uk (M.N.R.); 2 Departamento de Ecologia, Zoologia e Genética, Instituto de Biologia, Campus Universitário Capão do Leão, Universidade Federal de Pelotas, Pelotas 96010-900, RS, Brazil; rafael.kretschmer@ufpel.edu.br; 3 L. K. Ernst Federal Research Centre for Animal Husbandry, Dubrovitsy, 142132 Podolsk, Moscow Oblast, Russia

**Keywords:** avian genome, avian karyotype, interchromosomal rearrangements, intrachromosomal rearrangements, macrochromosomes, microchromosomes

## Abstract

Birds (Aves) are the most speciose of terrestrial vertebrates, displaying Class-specific characteristics yet incredible external phenotypic diversity. Critical to agriculture and as model organisms, birds have adapted to many habitats. The only extant examples of dinosaurs, birds emerged ~150 mya and >10% are currently threatened with extinction. This review is a comprehensive overview of avian genome (“chromosomic”) organization research based mostly on chromosome painting and BAC-based studies. We discuss traditional and contemporary tools for reliably generating chromosome-level assemblies and analyzing multiple species at a higher resolution and wider phylogenetic distance than previously possible. These results permit more detailed investigations into inter- and intrachromosomal rearrangements, providing unique insights into evolution and speciation mechanisms. The ‘signature’ avian karyotype likely arose ~250 mya and remained largely unchanged in most groups including extinct dinosaurs. Exceptions include Psittaciformes, Falconiformes, Caprimulgiformes, Cuculiformes, Suliformes, occasional Passeriformes, Ciconiiformes, and Pelecaniformes. The reasons for this remarkable conservation may be the greater diploid chromosome number generating variation (the driver of natural selection) through a greater possible combination of gametes and/or an increase in recombination rate. A deeper understanding of avian genomic structure permits the exploration of fundamental biological questions pertaining to the role of evolutionary breakpoint regions and homologous synteny blocks.

## 1. Avian Biology and Its Importance

With around 10,500 extant representatives, birds are the most species-rich of tetrapod vertebrates [1]. Modern birds belong to the phylogenetic Class Aves and the subclass Neornithes. They are characterized by a combination of features not seen together in other vertebrates including homeothermy, flight (except for penguins, ratites and some others who have lost the ability), oviparity, nesting, the presence of a beak (without teeth), a high metabolic rate, feathers, and a lightweight skeleton. Occupying almost all terrestrial and many aquatic habitats, birds have adapted to a range of climate extremes from inland Antarctica to the tropics. The highest levels of species diversity is seen in tropical regions [2]. The phenotypic diversity seen in birds is extraordinary; sizes range from the bee hummingbird (*Mellisuga helena*) at approximately 5 cm in length to the ostrich (*Struthio camelus*), which stands at over 2 m tall. Birds have a high core body temperature (39–41 °C), high blood glucose levels, and energy expenditure levels that are five or more times higher than those commonly seen in mammals. Comparisons with similar sized mammals show that birds tend to live longer, despite the higher energy use [3]. Birds are social, with varying degrees of communication complexity including the use of calls and song (in some cases communicating by visual display); they can be socially cooperative, exhibiting behaviors such as flocking and mobbing. Most birds also provide an extended period of parental care that is often shared between parents and/or with other birds [4,5,6].

Birds are critical to agriculture (both meat and eggs) and are also model organisms for studies of virology, immunology, and developmental biology, e.g., [7]; they can also be valuable companion animals for humans. From an evolutionary point of view, the Aves Class is the only extant example of the Dinosauria (Theropoda) clade, e.g., [8], sharing a common ancestor with mammals around 310 million years ago (mya). In addition, approximately 1400 extant birds (>10%) are currently listed as threatened with extinction, with over 160 species becoming extinct in the last 500 years [9]. Many of these extinctions are considered to be a result of anthropogenic climate and habitat change, in particular due to the introduction of alien species such as rats into island habitats [9]. Further understanding of birds from an evolutionary point of view is therefore crucial to understand vertebrate evolution and to protect current species from further risk.

## 2. Avian Evolution

Originating around 150 mya in the late Jurassic, birds evolved from a theropod dinosaur lineage [10,11] at a time when the supercontinent Pangaea was separating into two landforms (Laurasia and Gondwana). The fossil *Archaeopteryx lithographica* dates back to 150 mya, was found in the 19th century in late Jurassic limestone in Germany [12], and provides evidence of a transitional species between ancient dinosaurs and modern birds. Although previously considered to be the fossil representative of an early modern bird, features such as a bony tail and teeth rule out *A*. *lithographica* from being considered a true avian ancestor [13].

The oldest unambiguous fossil representative of Neornithes (modern birds), *Vegavis,* is an aquatic bird classified within Anseriformes and most closely related to Anatidae—ducks, geese, and swans [14]. Dating back to ~67 mya, the discovery of this fossil supports the notion that representatives of modern birds were co-extant with non-avian dinosaurs prior to the Cretaceous–Paleogene (K-Pg) boundary 66 mya [14]. The inherent difficulties in fossil dating due to geographic and depositional sampling bias have led to much controversy in the field of paleontology [10], meaning that analyses at a genomic level are a useful complement to a fossil record that may imperfectly represent actual avian ancestors. As a result, the dinosaur ancestor of birds is generally considered to be bipedal, terrestrial, and relatively small (small size being a pre-adaptation to flight) with a limited flying ability, not dissimilar to the Galliformes [15].

The study of genomics has revolutionized avian phylogenetic studies and, until the publication of a revised avian phylogeny by Jarvis et al. [16], the timing of avian diversification was the subject of much debate. The first avian divergence is now considered to have taken place around 100 mya when the Palaeognathae (ratites and tinamous) diverged from the Neognathae (Galloanserae and Neoaves). Within the Palaeognathae, the ratites and tinamous then diverged 84 mya, while the Neognathae diverged into its stem lineages, the Galloanserae and Neoaves, 88 mya. The Galloanserae divergence into the Galliformes (landfowl) and Anseriformes (waterfowl) occurred around the time of the K-Pg extinction event 66 mya, with major divergences of the Neoaves into Columbea and Passerea now dated to before the K-Pg boundary (67–69 mya). The rest of the divergences within Neoaves were largely complete at the ordinal level by 50 mya, with the Passeriformes basal split estimated to have occurred approximately 39 mya [16]. The K-Pg event was a period of abrupt, mass global extinction and extreme climate change, coinciding with the Chicxclub asteroid impact in Mexico [17], extremely significant for archaic birds (Ornithurae), of which the Neornithes are descendants. Recent fossil evidence points to a major radiation of advanced ornithurines occurring prior to the end of the Cretaceous period. The same group then suffered an abrupt extinction around the K-Pg event, with their disappearance from the fossil record from the Paleogene period onwards [18]. Genomic data from Jarvis et al. [16] also suggest that the K-Pg transition period was one of rapid Neornithine speciation, with 36 lineages radiating over a period of 10–15 million years. Jarvis et al. propose that these revised dates challenge some of the previously held assumptions that Neornithine lineages diversified explosively significantly after the K-Pg boundary [16].

This review is devoted to a specific aspect of genomics, “chromosomics”—the study of genomes from the perspective of the chromosome constitution or karyotype. The karyotype can be considered a low-resolution representation of the whole genome of a eukaryotic organism. Here, we provide a comprehensive, contemporary synthesis of data derived from a comparative analysis of the evolution of bird chromosomes, employing both chromosome painting and the use of BAC (bacterial artificial chromosome) technology. The insights presented and discussed herein are poised to emerge as a highly useful reference and source of information, offering substantial contributions to the field of avian comparative cytogenetics and genomics (“chromosomics”) resources for future studies.

## 3. Defining the Avian Karyotype

The karyotype of any eukaryote essentially defines its overall genomic structure. It allows gross genomic differences to be compared between species, ultimately building an evolutionary tree of gross genomic changes. Defining the avian karyotype in molecular cytogenetic terms is however notoriously difficult, largely due to the presence of a (usually) large number of morphologically indistinguishable microchromosomes [19]. Classification of the larger macrochromosomes (up to chromosome 9, including the sex chromosomes) is possible using classical cytogenetic techniques such as standard karyotyping but, beyond this size, it is near impossible to complete for most species—hence the publication of partial, rather than full, avian karyotypes in all cases apart from chicken [20]. Even at the macrochromosomal level, chromosome banding can be difficult to identify, thereby making a robust analysis of cross-species homology difficult and unreliable. The development of chromosome paints derived from the amplification and fluorescence labelling of chicken macrochromosomes has improved on this limited resolution and led to the publication of cross-species analysis (“zoo-FISH”) data including approximately 120 avian species from 22 different orders [19]. These studies are, however, restricted to analysis of the macrochromosomes. Some success has been achieved using microchromosomal chromosome paints, e.g., [21]; but, again, this is limited, largely due to the inability to separate each microchromosome by flow cytometry—the starting point for the generation of chromosome paints. A degree of success using a cross-species BAC mapping approach was originally reported, although this was limited to closely related species, with 70% success rates reported using chicken BACs on turkey (*Mealeagris gallopavo*) [22] reducing to under 40% when tested on duck (*Anas platyrynchos*) [23]. Marginally higher rates between chicken and duck have been reported elsewhere, however [24]. Up until the earliest years of the millennium, the apparent highly conserved nature of microchromosomes proved difficult to investigate using either classical or molecular cytogenetic methods, e.g., [22,23].

## 4. Avian Karyotypic Diversity

The highly distinctive, ‘signature’ avian karyotype is typically divided into around 10 macrochromosome pairs and around 30 pairs of evenly sized, morphologically indistinguishable microchromosomes [20,25,26,27,28,29,30,31]. The morphological similarity of the microchromosomes, and the sheer number of them, makes a full classical karyotype almost impossible to generate and analyze. In fact, although over 1000 karyotypes [19] have been published to date for a class that represents around 10,500 extant species, these are partial at best, with only 5–10 pairs of chromosomes easily identifiable. Rare exceptions to this ‘avian style’ signature karyotype include those with an unusually small diploid number such as the stone curlew (Charadriiformes, *Burhinus oedicnemus*; 2*n* = 42) [32] and the beach thick knee (Charadriiformes, *Esacus magnirostris*; 2*n* = 40) [25]; and those with an uncommonly high diploid number such as the kingfishers (Coraciiformes, *Alcedo atthis*; 2*n* = 132) [33] and hoopoes (Bucerotiformes, *Upupa epops*; 2*n* > 120) [25]. It is essential to note however that these deviations are not necessarily uniformly representative of their entire avian orders (e.g., Charadriiformes, Coraciiformes, and Bucerotiformes) (reviewed in [21]). Therefore, exceptions are not the rule within these orders. For instance, Ciconiiformes (storks), Pelecaniformes (ibis, herons, pelicans, hamerkop, and shoebill), Falconiformes (falcons), and Psittaciformes (parrots) usually exhibit lower diploid numbers (reviewed in [19]) but patterns differ, while toucans (e.g., *Ramphastos toco*; 2*n* = 114) defy the norm with usually higher diploid numbers [34].

At a molecular level, microchromosomes are particularly unique in being extraordinarily GC-rich and gene-dense, whilst accounting for only 23% of the genome but 48% of the genes [35,36,37,38]. Notably, in birds and snakes, microchromosomes also display a low transposable element content and high rates of recombination [39,40,41]. Burt [38] proposed that the microchromosomes present in birds were established in the ancestral vertebrate karyotype 400 mya. This appears to be supported by Nakatani and colleagues’ [42] study, which found that many avian microchromosomes corresponded directly with gnathostome ancestor protochromosomes. In turn, this implied that the characteristic avian karyotype was established at an extraordinarily early stage of evolution (see Section 9).

## 5. Sex Chromosomes in Birds

Unlike mammals, birds exhibit the highly conserved ZW sex chromosome system, with females being heterogametic (ZW) and males homogametic (ZZ) [43,44]. In all Neognathae, the Z and W chromosomes are differentiated in terms of size and morphology, with the W being largely heterochromatic, gene poor, and significantly smaller than the Z [45,46,47]. Exceptions to this rule include a few cases reviewed in Schartl et al. [48], where the W chromosome is heterochromatic and the same size as, or even bigger than, the Z chromosome. Ratites, however, have a W chromosome of a similar size to the Z and it is homologous in its entirety with the exception (in the case of emus) of a small region near the centromere [49,50,51]. It has been suggested that the alteration of chromatin conformation induced by transposable element (TEs) accumulation comprises an important early step in sex chromosome differentiation [52]. Despite the difference in size, it can be inferred that the ZW system was present prior to the divergence of the Palaeognathae and Neognathae lineages [53], but that the differentiation in size between the two chromosomes occurred afterwards. Although superficially resembling the XY system seen in mammals, the XX/XY (mammalian) and ZZ/ZW (avian) systems exhibit no homology [54] and have completely independent origins. The avian Z chromosome shares homology with human autosomes 5, 9, and 18 [55]. The human/mammalian X chromosome, on the other hand, shares homology with a block of the q-arm of chicken chromosome 1 and a 20 Mb portion of the p-arm of chicken chromosome 4 (a microchromosome in most other birds) [56]. The sex-determining gene in birds is not SRY as in mammals (the homologue of which in fact lies on chicken chromosome 4) [57]. Instead, it has been suggested that the gene *DMRT1* found on the Z chromosome may be the key to sex determination using a dosage-dependent system. Male determination requires two copies of the gene as found in ZZ males, and *DMRT1* has also been shown to be required for testis formation [58]. There is still much debate, however, as to what determines sex in birds, with possible candidates (among other theories) including W-specific genes that may determine ovarian function [57]. Improvements in the assembly of the Z chromosome have been achieved using a BAC-based approach [55], along with further work to improve the assembly of the W chromosome [59].

Recent studies revealed a surprising dynamism in avian sex chromosomes, challenging the earlier perception of their stability. One remarkable discovery involved the identification of a multiple-sex chromosome system (♂Z1Z1Z2Z2/♀Z1Z2W) in the penguin species *Pygoscelis adeliae* (Sphenisciformes) [60]. Additionally, instances of independent autosome–sex chromosome fusions have been identified in Sylvioidea species through the analysis of genomic data [61]. Moreover, neo-sex chromosomes have been identified in parrots [62], while similar findings were observed in a cuckoo species [63]. These findings collectively highlight a previously unrecognized diversity and dynamism within avian sex chromosome systems.

## 6. Chromosomal Rearrangements in Birds

As described above, a key feature unique to birds is the high level of karyotypic stability. That is, the majority (~70%) of avian species have a karyotype that is very similar in terms of size and gross genomic structure to that of the chicken (2*n* = 78). Exceptions to this rule include the Ciconiiformes (storks), Pelecaniformes (ibis, herons, pelicans, hamerkop, and shoebill), Falconiformes (falcons), and Psittaciformes (parrots), which have lower diploid numbers than chickens, fewer microchromosomes, and thus evidence of chromosomal fusion (reviewed in [19]). The use of chromosome paints derived from chicken flow-sorted chromosomes demonstrated a high degree of conservation between the macrochromosomes. This supports the view that the avian genome structure is highly conserved, even across large phylogenetic distances. Technical difficulties creating microchromosomal paints have limited the scope of their use for this analysis. Recently, a series of papers using a microchromosomal BAC-based FISH approach found evidence of microchromosomes fusing to macrochromosomes in a few avian orders, thereby filling in gaps that had been unassigned, e.g., [21,64].

In contrast to mammals, birds therefore exhibit a slow rate of change in *inter*chromosomal rearrangements [65,66,67]. Despite this apparently slow rate, it is likely that the same does not apply to *intra*chromosomal rearrangements, which are seen considerably more frequently [68,69,70]. A comparison of the genomes of the chicken, turkey, and zebra finch and analysis using the Genalyzer tool [71] revealed a high degree of intrachromosomal rearrangement within the macrochromosomes, many of which were subsequently confirmed by FISH. Analysis of intrachromosomal rearrangements in the microchromosomes, however, has been limited to a few studies and few avian orders. The first investigation by Rao et al. [72] used the radiation hybrid method to assemble the duck genome and also compared the microchromosomes of the duck to those of the chicken. A second study by Lithgow et al. [21] found no interchromosomal rearrangements between chicken, turkey, and zebra finch microchromosomes, but found multiple intrachromosomal changes.

As well as facilitating characterization of the chicken karyotype [20,37], the generation of chromosome paints for chicken chromosomes 1–9 plus Z and W led to a surge in avian comparative genomics research, e.g., [49,73,74,75,76,77]. A high degree of success has been accomplished using these paints, with results achievable in species as evolutionarily far-removed from chicken as falcons, ostriches, and emus [49,77,78]. A summary of the avian species investigated using chicken chromosome paints is listed in Table 1 and Supplementary Table S1.

**Figure 1:**
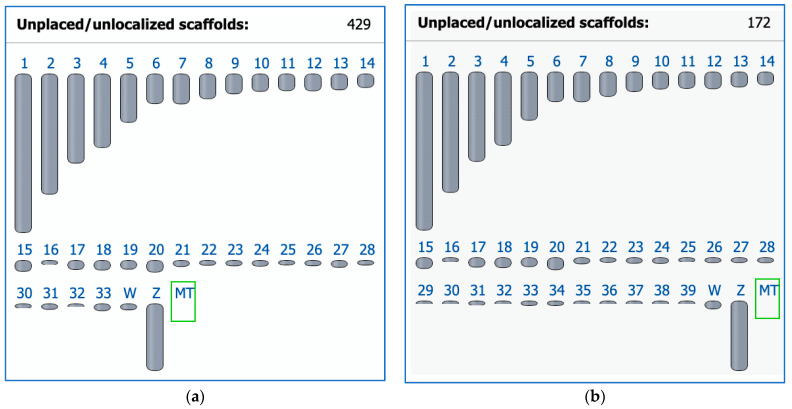
Ideogram view in the NCBI Genome Data Viewer for two chicken (*Gallus gallus*, GGA) representative genome assemblies. (**a**) The previous assembly GRCg6a (GCF_000002315.6) released on 27 March 2018 with 1 to 33 autosomes. (**b**) The latest assembly bGalGal1.mat.broiler.GRCg7b (GCF_016699485.2) as of 19 January 2021 has 1 to 39 autosomes (including macrochromosomes GGA1–GGA9 and microchromosomes GGA10–GGA39) and fewer unplaced scaffolds as a result of work later published in Huang et al. [133]; Z and W, sex chromosomes; MT, mitochondrial genome.

In 2004, the chicken became the first avian species for which a whole genome sequence was generated [130] and a karyotype fully defined [20]. At that time, chromosome paints were generated from microdissected metaphase preparations to identify all chromosomes uniquely. Subsequent efforts to sequence DNA from these clones, however, proved unsuccessful [131]. Moreover, the original chromosome paint probes from Masabanda et al. [20] have since degraded [131]. Reliable tools for the detection of these chromosomes were developed that contributed to identifying chicken microchromosome syntenies across many avian groups [132]. Indeed, until recently, the very smallest of the microchromosomes, the ‘D group’ (chromosomes 33–39) [20], had no sequences associated with them in the genome assembly (Figure 1a). At the time of writing, all chicken macro- and microchromosomes now have respective sequences assigned to them and are annotated thanks to the recent work of Huang et al. [133], although there are still 172 scaffolds to be placed or localized (Figure 1b). The chicken chromosome-level assembly is the most widely used reference genome in the avian comparative genomic field for the cytogenomic and phylogenomic analyses of birds.

Unlike research performed on mammalian chromosomes, hybridization across a greater evolutionary distance (i.e., beyond the phylogenetic Class) is possible with chicken chromosome paints. For example, homology has been detected between chicken, turtles, and crocodiles, all of which last shared a common ancestor over 250 mya [134,135]. The use of microchromosomal paints, however, has been comparatively limited [32,49,92], largely due to the paints being divided into ‘pools’ of microchromosomes rather than being assigned to separate, entire chromosomes [21]. Whilst able to define whole blocks of homology between species, the orientation of the blocks cannot be defined using chromosomes nor can intrachromosomal rearrangements between species. To overcome both limitations, a BAC-based approach was necessary, either in conjunction with chromosome paints or in isolation.

Chromosome painting studies (zoo-FISH) identified several avian species that (like the chicken) exhibit a fusion of ancestral avian chromosomes 4 and 10, as listed in Table 2, and hence show examples of possible homoplasy. In most other species analyzed, including all representative Paleognathae species investigated thus far, the two chromosomes appear separately. This example is of particular interest because the ancestral chromosome 4 (chicken chromosome 4 q-arm) is highly conserved and syntenic with human chromosome 4, meaning that it must be present in the common ancestor 310 mya [136]. In addition, the p-arm of chromosome 4 is orthologous to an ancestral microchromosome, with zoo-FISH evidence of interstitial telomere signals next to the centromere. It also seems that this ancestral region has not lost the characteristically microchromosomal properties of high gene density and recombination rate [130]. The repeated pattern of this rearrangement across multiple species may, of course, be an example of homoplasy and therefore the result of numerous independent fusions, or perhaps an interesting example of hemiplasy.

## 7. Closer Examination of Avian Microchromosomes

As described above, most of the avian comparative genomic studies performed to date at a chromosomal level have been limited to investigating macrochromosomal chromosome painting because microchromosomal paints were not available. The development of BAC libraries as a product of genome sequencing, e.g., [132,133,137] has, however, facilitated the development of a set of BACs that have been selected that successfully hybridize to chicken microchromosomes. Using BACs derived from the (sequenced) microchromosomes in combination with the macrochromosomal chicken paints therefore allows molecular cytogenetic examination of almost the entire chicken genome and its chromosomal homologs in other species.

Identification of the genomic features unique to these BACs using a bioinformatic approach [132,138] led to a refinement in the methods used to select BACs designed to hybridize across multiple species [8,70,114,132,139,140]. This resulted in an improvement in hybridization rates between species by several orders of magnitude. Selection of the BACs was based on successful hybridization across five core avian species [70,132] and by the position of each BAC in the reference species (at the most distal region of each chromosome). These BACs provided a consistent anchor point from which to compare species to track chromosomal rearrangements over time. As reported by Damas et al. [132], rather than being limited to comparing multi-species’ chromosomal rearrangements within a specific order, this approach allowed for comparison across an entire Class (and, to some degree, even beyond this [132,138]).

**Figure 2:**
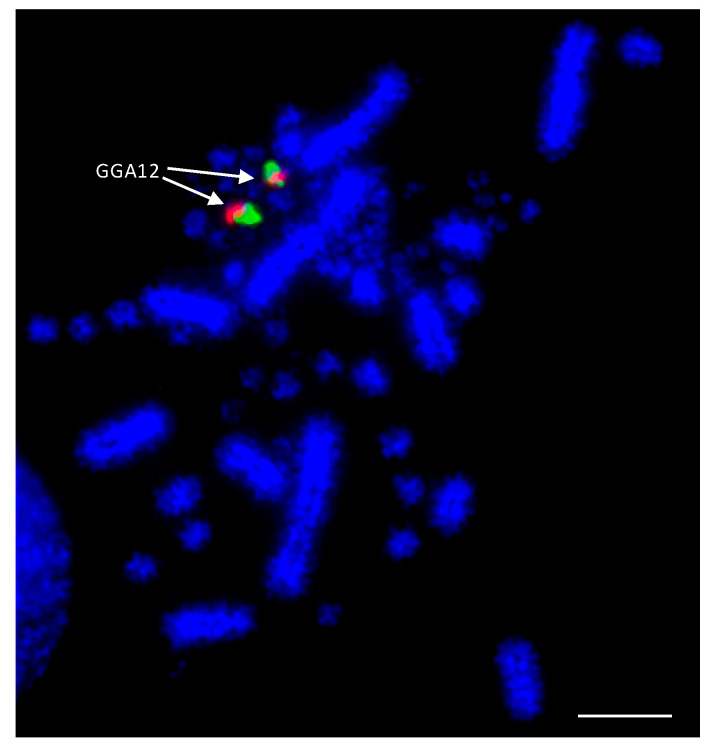
Example of dual FISH results for chicken (*Gallus gallus*—GGA) chromosome 12 to confirm correct mapping. The p-arm BAC (CH261-88K1) is labelled with FITC (fluorescein isothiocyanate) (green) and the q-arm BAC (CH261-152H14) is labelled with Texas Red (red). Scale bar 10 μm.

As the best-characterized avian species with the largest number of BAC libraries available, e.g., [139], the chicken was used as the reference avian species in most of the studies. BACs located in the most distal (where possible) regions of the p- and q-arms of chromosomes 1–28 were selected according to the selection criteria described by Damas et al. [132]. The co-hybridization of p- and q-arm BACs for each chromosome was performed to verify correct mapping of the BACs, producing bright punctate signals for each of the chicken chromosomes, an example of which is shown for chicken chromosome 12 (GGA12) in Figure 2.

In order to assess whether gross chromosomal rearrangements occurred between a range of phylogenetically distant species, the probes were tested on 40+ different species as listed in Table 3 and Supplementary Table S2.

Clear, punctate signals (similar to those seen on the chicken metaphases) were achieved for all microchromosome BACs for each species (apart from the two BACs for chicken chromosome 25 when tested on Passeriformes representatives that did not produce a signal). Clear signals were achieved for all macrochromosome BACs with a few individual species-specific exceptions.

**Figure 3:**
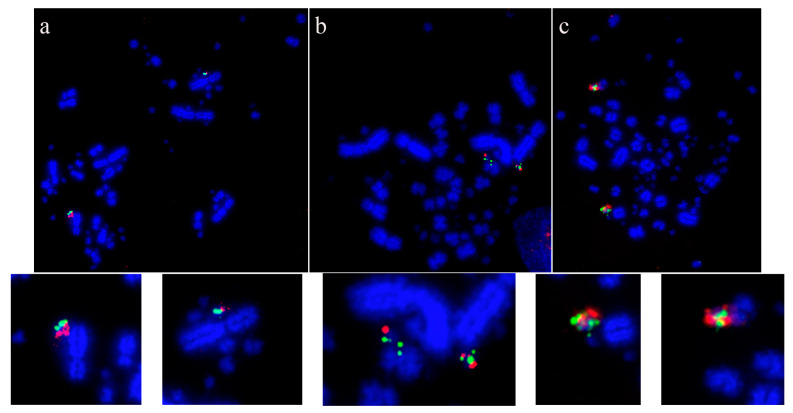
Microchromosomal conservation observed across a wide range of avian species as revealed by testing BACs from chicken chromosome 24 (CH261-103F4 FITC in green and CH261-65O4 Texas Red in red): *Phalacrocorax brasilianus* (**a**), *Crotophaga ani* (**b**), and *Geotrygon montana* (**c**). Frame enlargements immediately beneath, occasional multiple signals (e.g., in (**b**,**c**)) as commonplace in FISH experiments and representing small amounts of background hybridization.

Using this set of cross-species probes, specifically to investigate the microchromosomes, permitted analysis at a higher resolution than previously achieved. In most studies, two BACs were selected from each of the sequenced chicken microchromosomes (from GGA10 to GGA28, except for GGA16) and dual FISH was performed on a total of 42 avian species (Table 3). In all species tested, regions homologous to chicken chromosomes 22, 24, 26, and 27 appear to have remained intact as entire microchromosomes with no evidence of chromosomal fusion [64,140]. Figure 3 shows representative images for chicken chromosome 24 tested on multiple species with the BACs illustrating that this chromosome appears to remain intact as a microchromosome in all species tested with no sign of interchromosomal rearrangement.

### 7.1. Species with No Apparent Interchromosomal Rearrangement between the Microchromosomes

**Figure 4:**
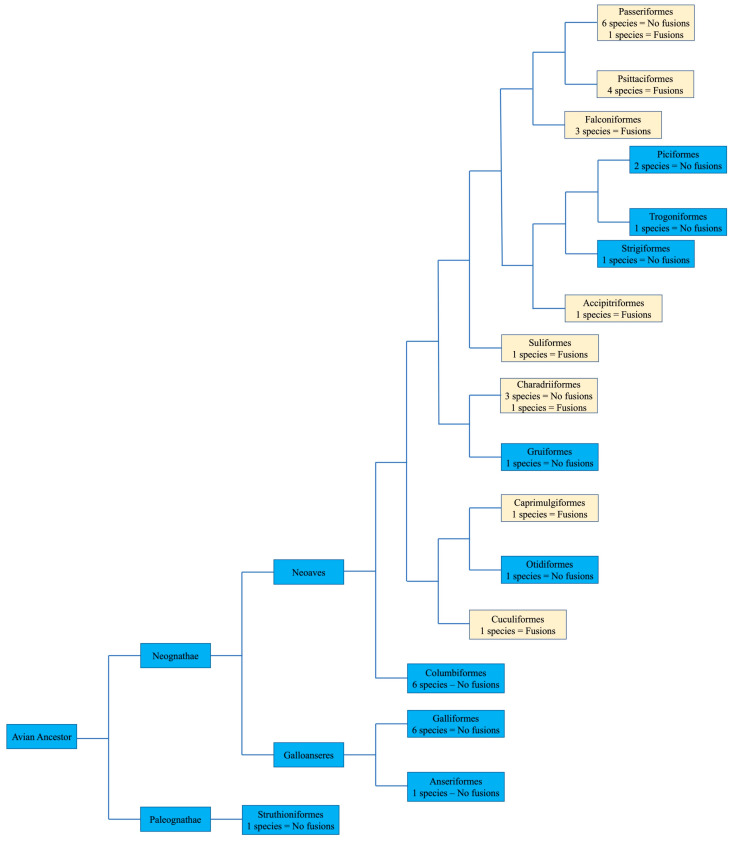
Bird phylogeny illustrating the presence or lack of interchromosomal rearrangement involving microchromosomes based on BAC FISH. The numbers of species with or without interchromosomal rearrangement involving microchromosomes is illustrated in each order. Macrochromosomal fusions are not listed. Phylogenetic relationships followed Jarvis et al. [16]. Light colored boxes indicate where interchromosomal changes occurred.

No apparent changes from the ancestral microchromosomes appear to have occurred among the representatives from the following orders: Galliformes, Gruiformes, Anseriformes, Columbiformes, Otidiformes, Piciformes, Trogoniformes, Strigiformes, and Struthioniformes (Figure 4). The microchromosomes of each bird remain conserved in the same pattern exhibited in the chicken with BACs hybridizing consistently together across all species tested despite there being over 100 million years since they diverged.

### 7.2. Species Demonstrating Rearrangement between Microchromosomes

Herein, we review the main findings in the orders with extensive chromosomal reorganization. BAC FISH results indicated fusions involving microchromosomes in representative species of Psittaciformes, Falconiformes, Caprimulgiformes, Cuculiformes, Suliformes, and Passeriformes (Figure 4). We also discuss the karyotype of Ciconiiformes and Pelecaniformes, as chromosome painting with chicken and stone-curlew chromosome paints also indicated extensive fusion events in members of these orders.

#### 7.2.1. Psittaciformes

**Figure 5:**
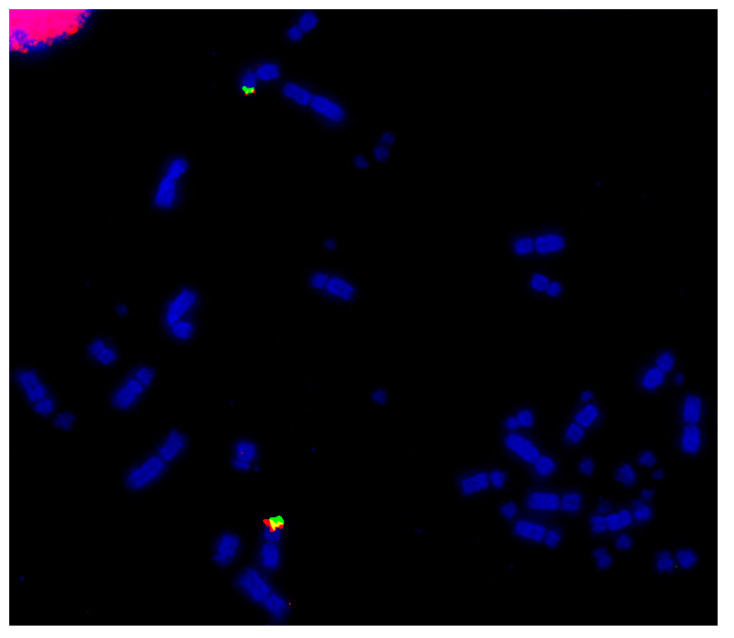
Hybridization of GGA22 BACs (CH261-40J9-FITC in green and CH261-18G17-Texas Red in red) to monk parakeet (*Myiopsitta monachus*, MMO) metaphase illustrating fusion of the ancestral microchromosome to a macrochromosome.

Among the Psittaciformes, four species have been investigated using microchromosome BACs probes: the kakariki (*Cyanoramphus novaezelandia*), the cockatiel (*Nymphus hollandicus*), the budgerigar (*Melopsittacus undulatus*), and the monk parakeet (*Myiopsitta monachus*). Shared rearrangements were not observed between these species [64,119,138]. Fusion events were detected for the homologs for GGA10, 11, and 14 in the kakariki, the cockatiel, and the budgerigar with the additional fusion observed of the GGA13 homolog in the budgerigar. The monk parakeet showed several tandem fusions between microchromosomes and fusions between macrochromosomes and microchromosomes, resulting in a karyotype with the low diploid number of 48 [119]. An example of the microchromosome fusion in the monk parakeet genome is shown in Figure 5.

**Figure 6:**
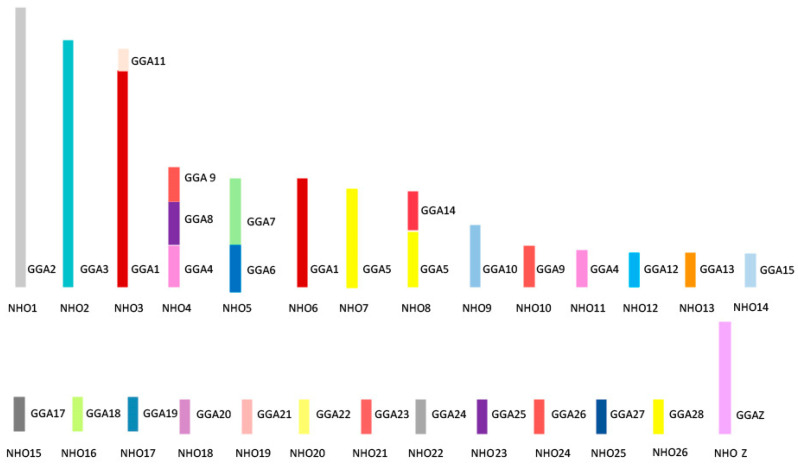
Ideogram representing karyotypic structure of the cockatiel (*Nymphus hollandicus*, NHO) illustrating an overall structure. Each chicken (GGA) homolog is represented as a different color.

Figure 6 shows the overall karyotypic structure of the cockatiel and illustrates that, despite broadly similar patterns of rearrangement, there are fewer rearrangements that have occurred between the macrochromosomes when compared to the budgerigar. The kakariki karyotype appears most similar to the budgerigar but requires further mapping to confirm the overall structure as there are no previously published studies for this species. Macrochromosomal rearrangements are based on those previously established through chromosome painting studies by Nanda et al. [115] and confirmed by BAC FISH.

#### 7.2.2. Falconiformes

**Figure 7:**
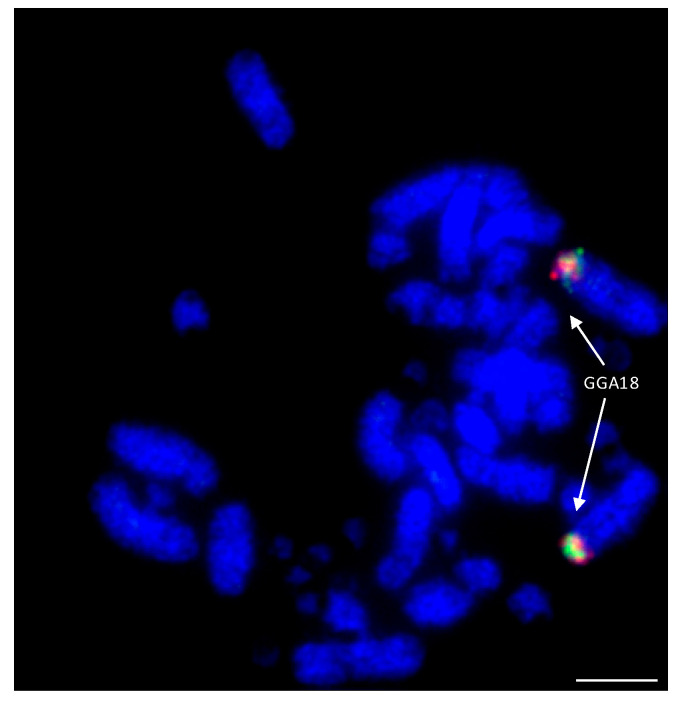
Hybridization of GGA18 BACs (CH261-60N6-FITC in green and CH261-72B18-Texas Red in red) to peregrine falcon (*Falco peregrinus*) metaphases illustrating fusion of ancestral microchromosome to a macrochromosome. Scale bar 10 μm.

Among the Falconiformes, extensive rearrangement appears to have taken place with regions homologous to GGA microchromosomes 10, 12, 13, 14, 15, 17, 18, 19, 20, 21, 23, and 28 fused to GGA macrochromosome regions [114,138]. An example of this is illustrated in Figure 7 where GGA18 homologs are fused to a macrochromosome in the peregrine falcon. Lineage-specific rearrangements were apparent with no evidence of chicken chromosome homologs 15, 18, 19, 23, and 28 being rearranged in any of the other (non-falcon-related) species tested. Interestingly, 15, 18, and 19 appear to have fused together as one chromosome (to chicken homolog 4) in both falcon species tested, while 23 and 28 have both fused to the homolog of chicken chromosome 2, which, at some point (either pre- or post-fusion) has split into two chromosomes. Both of the falcon species tested (peregrine and saker) appear to exhibit the same pattern of rearrangement (with the exception of peregrine chromosome 1 for which there is a centric fusion), suggesting that any lineage-specific rearrangements rapidly became fixed in the population with little interchromosomal rearrangement since. In addition, there appears to be no interchromosomal rearrangement between each pair of BACs tested, suggesting that these regions of DNA are highly conserved and not prone to breakage.

**Figure 8:**
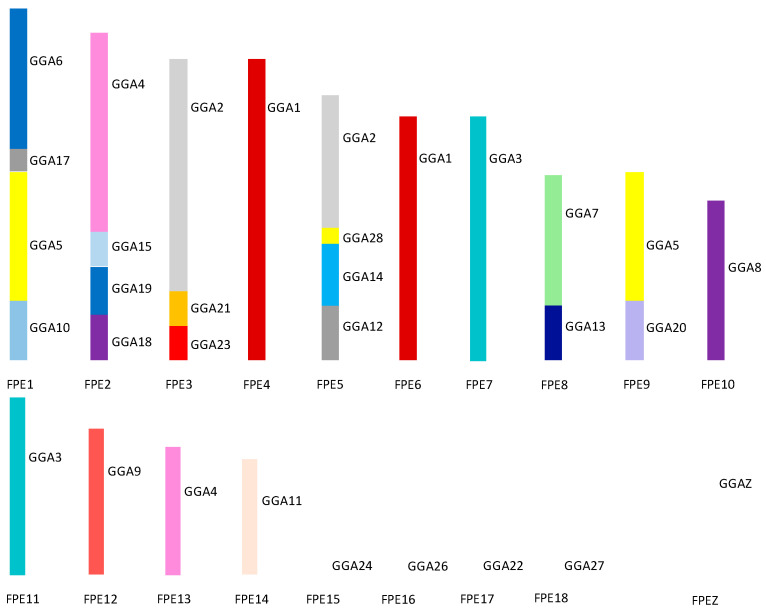
Ideogram representing overall karyotypic structure of the peregrine falcon (*Falco peregrinus*—FPE) illustrating an extensive amount of interchromosomal rearrangement throughout the karyotype. Each chicken (GGA) homolog is represented as a different color.

Figure 8 illustrates the overall karyotype structure of the peregrine falcon tested showing extensive interchromosomal rearrangement between the micro- and the macrochromosomes [114,138]. The karyotype of the saker falcon follows the same pattern with the exception of peregrine chromosome 1 [138]. In the saker falcon, this chromosome has split into two chromosomes with the breakpoint occurring within the region of the GGA5 homolog. This suggest that this is a fission that has occurred after the falcon karyotype was formed rather than a later peregrine-specific fusion.

#### 7.2.3. Ciconiiformes

Most of the reports about the chromosome organization of storks (Ciconiiformes) have relied solely on analyses by conventional staining; however, an interesting variation in the diploid chromosome number was found (2*n* = 52 to 78) [reviewed in 21]. Considering that most species have similar macrochromosomes, some authors propose that karyotype evolution mainly involves fusions between microchromosomes, which were later confirmed by chromosome painting [98]. Nevertheless, the exact microchromosomes involved in the rearrangements were not identified and this question therefore warrants additional studies.

#### 7.2.4. Pelecaniformes

Molecular cytogenetics is still scarce in this order; however, recent studies using chromosome painting with chicken and/or stone curlew (*B*. *oedicnemus*, BOE) paints indicated that the karyotype of Pelecaniformes is reorganized, similar to that of the parrots and falcons [99,100]. The stone curlew chromosome painting in three Pelecaniformes species (*Ardea cinerea*, *Egretta garzetta*, and *Nipponia nippon*) indicated that different chromosome rearrangements occur in different Pelecaniformes lineages [99]. The main rearrangements were fusion events, including macro- and microchromosome. In *Syrigma sibilatrix,* the GGA8, GGA9, and GGA10 chromosome paints hybridized to the long arms of biarmed macrochromosomes, also indicating fusions with microchromosomes [100]. Although BOE and chicken (GGA) microchromosome paints do not allow for the identification of the exact microchromosome involved in the fusion events, these studies indicated that fusion events involving these tiny chromosomes are nonetheless frequent among Pelecaniformes. Future studies using BACs probes are therefore necessary.

#### 7.2.5. Caprimulgiformes, Cuculiformes, Suliformes, and Passeriformes

While several studies demonstrated that interchromosomal rearrangements involving macro- and microchromosomes have played a role in the karyotype evolution of Ciconiiformes, Falconiformes, and Psittaciformes, recent studies have also observed this type of rearrangement in Caprimulgiformes, Cuculiformes, Suliformes, and Passeriformes species [140,142]. However, in these studies, only one representative species was investigated (Caprimulgiformes, Cuculiformes, and Suliformes) or the rearrangements were found only in one species (Passeriformes). Future studies are therefore necessary to explore whether these fusions are species-specific or are a common feature of each order. Most species of Passeriformes demonstrate the typical avian karyotype, as evidenced using microchromosomal BAC FISH, where only one species (*Tolmomyias sulphurescens*, 2*n* = 60, Rhynchocyclidae) from seven demonstrated microchromosomal fusions [142]. It is likely that the low diploid number is specific to the Rhynchocyclidae family, while remaining conserved in other Passeriformes species [142].

Among the Cuculiformes, *Crotophaga ani* is the only species to date to be investigated using BAC FISH, with extensive chromosome reorganization involving macro- and microchromosomes observed. A fusion between chicken chromosome 17 and Z was also found. Z–autosome Robertsonian translocations are rare in birds and have only been otherwise observed in Sylvioidea species (Passeriformes) [61,146,147,148] and in selected parrot species with a genome sequence [62].

## 8. Microchromosomes and their Conservation, Whether Discrete or Fused

Microchromosomal rearrangement has long been considered to occur rarely compared to other chromosomal rearrangements in birds. These highly gene-dense chromosomes are thought to have changed very little throughout the last 100 million years of avian evolution [149] with a high degree of conservation potentially dating back even further to the ancestral vertebrate 400 mya [38,42]. Prior to the publication of cytogenetic studies in the last few decades however, there has been little cytogenetic evidence to support the notion of this degree of conservation. What evidence is available was originally focused largely on closely related and karyologically similar species such as chicken and duck [23,24]. The BAC-based approach enabled analysis across avian representatives from 17 different orders, all of which share a common ancestor over 100 mya. These results clearly illustrate an extraordinary level of microchromosome conservation, with 9 out of the 17 orders exhibiting no apparent change from the microchromosomal pattern exhibited in the chicken. From a microchromosomal point of view, these results support the hypothesis proposed in our previous study [150] that the avian ancestor most closely resembled the chicken. Of the avian species that did exhibit microchromosomal rearrangements, the three representatives of the Falconiformes (the saker, peregrine falcon, and gyrfalcon) share the same pattern of fusion, from which it can be inferred that early in the evolution of this order there was a large degree of rearrangement that became fixed in the population [114,138]. It may therefore be that there is some biological advantage to this karyotypic structure for these birds, perhaps due to the high metabolic demands required by birds of prey. Of the other highly rearranged order, the Psittaciformes, the microchromosomal fusions exhibited in each of the species are not consistent with one another. This suggests that karyotypic evolution has continued from their common ancestor and that species-specific rearrangements are apparent [64,138].

In all these cases, however, it appears that there is a bias towards the microchromosomes remaining as discrete units, even when fused into highly complex karyotypes such as those of the Falconiformes and the Psittaciformes. As mentioned above, this same pattern is evident in the chicken, where the p-arm of chromosome 4 is a microchromosome in most other species, and thus ancestral (see also Table 2). In the chicken, despite fusing to a macrochromosome, it remains intact, even retaining all its uniquely microchromosomal sequence characteristics such as a high GC and gene content [130].

Even considering these lineage specific rearrangements, there appear to be four microchromosomes (GGA22, 24, 26, and 27) that across all birds tested thus far remain conserved in their entirety, with no signs of apparent fusion. In the chicken, these are four of the smallest sequenced chromosomes with sizes ranging from 4 to 6 Mb. Further sequence analysis may reveal signature features of these chromosomes that may indicate a biological reason as to why these chromosomes are left intact. If there is any correlation with the size of the chromosomes and their lack of rearrangement, then this would suggest that the very smallest ‘D-group’ chicken microchromosomes (33–39) [20] are also less prone to chromosomal fusion. In fact, upon the exclusion of the two most rearranged lineages (parrots and falcons), a discernible pattern emerges where species from various orders appear to have accumulated primarily species-specific microchromosomal rearrangements rather than a shared characteristic. All other species analyzed exhibit the same pattern of conserved microchromosomal arrangement. Given that orders such as the parrots and the falcons are karyotypically the exception rather than the rule, this illustrates quite how profound this level of genome conservation really is.

In addition to the aforementioned attributes of microchromosomes, it is essential to highlight further distinctive features. Microchromosomes exhibit notable arrangements in the interior of the interphase nucleus, with macrochromosomes at the periphery [37,151,152,153]. Interestingly, studies of both avian and primate demonstrate that fusions involving gene-dense chromosomes with gene-poor ones do not appear to alter their nuclear positions [64,154,155]. A noteworthy characteristic is that microchromosomes also exhibit consistently high degrees of interchromosomal interaction (particularly with other microchromosomes), being co-localized in this central nuclear domain. This is observed across all microchromosomes in reptiles and birds [152,153], suggesting that this feature can be regarded as an ancestral trait. Interestingly, this persists even after their integration into a macrochromosome, albeit eroding over time [153]. Nevertheless, it is crucial to note that newly emerged microchromosomes swiftly establish high interactions with other microchromosomes [153], perhaps because they consist of a higher proportion of open chromatin compared to macrochromosomes. For a more comprehensive exploration of microchromosome properties, refer to the in-depth review by Srikulnath et al. [156].

## 9. Dinosaurian Origins of the Signature Avian Karyotype

Although the signature avian karyotype contains both macro- and microchromosomes, it would be wrong to suggest that the presence of both macro- and microchromosomes alone are a unique feature of avian genome organization. Indeed, microchromosomes are typical of most amniotes (many reptiles such as snakes, turtles, and lizards) with mammals and crocodilia (the only extant examples of non-avian archosaurs) being exceptions [157,158,159,160]. The greatest number and smallest size of microchromosomes are, however, typically found among birds. Burt [38] hypothesized that some microchromosomes were present in the common dinosaur ancestor that gave rise to birds (that probably had 2n = ~60) and that a series of fissions in the avian lineage resulted in the basic pattern of 2*n* = 80 (~30 pairs of microchromosomes) becoming fixed before the Palaeognathae–Neognathae divergence 100 mya. We, however, proposed that such a fixation may have been much earlier, at around 255 mya [8], with the basic pattern established in dinosaurs and pterosaurs and remaining relatively stable thereafter.

Evidence provided by a number of studies, e.g., [138,150,161] leads to suggested possible mechanisms why, with relatively rare exceptions, avian genomes remain evolutionarily stable interchromosomally and have possibly done so through dinosaur and pterosaur lineages. The absence of interchromosomal rearrangement either suggests an evolutionary advantage to retaining this signature avian/dinosaur/pterosaur configuration, or else little opportunity for change. Evidence of considerable intrachromosomal change in pigeons [69,70,132] and Passeriformes species [68,70,150] provides evidence that intrachromosomal change proceeds largely unhindered and can accelerate in line with rapid speciation events. Indeed, the near absence of interchromosomal rearrangement is no barrier to diversity and a direct correlation has been reported between the rates of speciation and intrachromosomal rearrangement [162]. There may even be an evolutionary advantage to maintaining a karyotypic structure formed of many compact, gene-rich microchromosomes [150].

It is a reasonable assumption that the characteristically stable avian gross karyotypic structure has a reduced opportunity for chromosome rearrangement, as there are low numbers of recombination hotspots, fewer repeat structures such as transposable elements, and fewer endogenous retroviruses. All of these genomic features have been previously demonstrated to provide substrates for interchromosomal rearrangement and all are sparser in avian, compared to other genomes [149]. In previous studies it has been argued that the signature avian karyotype evolved in response to the shrinking of the genome in birds as a result of the metabolic demands of flight [163,164]. The results reviewed here, however, indicate that the basic karyotype structure was in place long before avian genome size reduction. The average genome size in non-dinosaur and non-avian saurians (lepidosaurs, turtles, and crocodiles) is around 3 Gb [165] and is significantly smaller in saurischian (1.78 pg) in comparison to ornithischian dinosaurs (2.49 pg) [166]. Although flight evolution may be a factor in genome size reduction, therefore (pterosaurs are reported to have smaller genomes than other Avemetatarsalians [167] and bats have smaller genomes than other mammals [168]), other factors are clearly in play as flight only evolved in therapods approximately 150 mya [169]. It is possible therefore that the evolution of the karyotype was a driver of genome size reduction rather than the other way around.

## 10. Why Do Some Avian Genomes Break the Rules?

Explanations as to why some species such as falcons and parrots exhibit such a high degree of interchromosomal rearrangement, particularly microchromosomal fusion (see previous sections), when the majority do not, remain topics of debate. As mentioned above, there are other groups (such as kingfishers) that have an unusually high (2*n* = 130+) number of chromosomes [25]. Indeed, both higher and lower than usual deviations from the typical (2*n* = ~80) organization can occur in the same group, e.g., the Adélie penguin (2*n* = 96 in (males) and 95 (females)) [60] and the Magellanic penguin (2*n* = 68) [170]. This suggests that similar mechanisms can cause both a rapid reduction and a rapid increase in chromosome number. The short time period over which these changes occurred in the penguins and the rearranged karyotypes of the Falconiformes (but not the sister group Strigiformes) and the Psittaciformes (but not the sister group the Passeriformes) suggest that these changes can happen quickly in evolutionary terms. Vertebrates with large, repeat-rich genomes (such as mammals and amphibians) frequently demonstrate rapid intra- and interchromosomal rearrangements [171]. The results reviewed here suggest that birds too can undergo similar changes in certain groups although there is little evidence that these highly rearranged avian genomes are particularly large or more repeat-rich than other avian genomes. Zebra finch and budgerigar comparisons indicate a similarly high rate of chromosome mutation in both groups but these features appear to be a result of fixed interchromosomal rearrangements that have arisen due to the exploitation of evolutionary niches, while, in other avian species, such fixation is prevented resulting in maintenance of the signature avian karyotype. Why some become fixed, and others do not, is a relatively understudied field, although clues may lie in the study of gene ontology terms present in EBRs. Farré and colleagues found a correlation between EBRs and specific avian adaptive features in individual species, including forebrain development in the budgerigar, consistent with this species being not only a vocal-learner but having distinctive neuronal connections compared to other vocal-learners [172]. As more genomes become available with better assemblies, these analyses may well point to adaptive phenotypic features of individual orders and families.

## 11. Conclusions

Regardless of the exceptions to the rule, the fact remains that the signature karyotype described here is an evolutionarily successful one, and it has probably been in place for around 250 million years. This essentially means that therapod favorites such as the *T*. *rex* and *Velociraptor* probably carried an avian-like karyotype, unless they were one of the well described exceptions to the rule. The greater possible combination of gametes from having more chromosomes and the increase in overall recombination rate per chromosome (despite the lack of recombination hot spots) ultimately has the effect of generating variation, the driver of natural selection. Burt [38] suggested that a higher recombination rate has also contributed to the unique genomic features seen in microchromosomes such as a high GC content, low repeats, and high gene density which subsequently led to the maintenance of the typical avian karyotype. The potential for greater variation may therefore explain, in part, why there is incredible phenotypic diversity among all dinosaurs (including birds), why they were the dominant land vertebrate for hundreds of millions of years, and why, even then after the majority were wiped out by the K-Pg event 66 mya, they were able to diversify in a great adaptive radiation into a fantastically diverse class of organisms—neornithine birds [173].

Armed with the above information, there are fundamental biological questions relating to the significance of genomic regions prone to chromosomal breakage (EBRs) and regions of conserved synteny (HSBs) that can be addressed. For instance, why and how often do these rearrangements occur, and what advantage do these rearrangements (or lack of rearrangements) confer that are crucial to understanding patterns of evolution and speciation? For examples of such work, see the studies of Larkin, Lewin, and colleagues (some involving our own collaborations with them [8,64,70,158,174,175,176,177,178,179,180,181,182]). Given the oft-mentioned extraordinary phenotypic diversity seen in birds (and other dinosaurs) and the apparently paradoxical highly conserved nature of their overall genome structure, the tools developed and used in the studies described herein have started the process of answering some of these questions. The next obvious step in using this approach would be to extend the range of the avian species tested in order to assess whether these patterns herein are consistent across all orders, genera, and species. In addition, the preliminary work overviewed here using BACs with conserved synteny to identify chromosomal conservation and rearrangement in non-avian reptiles illustrates that these tools can also be used in other, even more phylogenetically distant clades. Moreover, since sequences have recently been assigned to the smallest “D-group” chromosomes (33–39), a similar approach may, in future, be employed to establish whether these highly elusive microchromosomes also exhibit similar patterns to those already identified.

Undoubtedly, new prospects in the study of avian genomes are opening up due to the introduction of the latest technologies that make chromosome-level genome assemblies achievable without necessarily involving chromosome preparations [183,184,185,186,187,188,189,190,191]. These are characterized by some recent mammalian genome chromosome level assemblies [189,190,191] and some more recent avian ones [191,192,193]. Technologies include long-read sequencing, optical mapping, and others [194,195,196,197], as well as de novo PacBio long-read and phased avian genome assemblies for complementing and rectifying reference genes previously produced using short and intermediate reads [198]. According to long-read genome assemblies, the number and structure of MHC loci in birds vary remarkably [199]. The possible influence of post-transcriptional regulation on the consequences of gene dosage on the avian Z chromosome can also be shown by single-molecule long-read sequencing [200], and optical mapping data have proved helpful in improving genome assembly, e.g., in the ostrich [201]. Using long reads, linked reads, and optical mapping, three divergent chromosomal regions with different inversions between migratory songbird phenotypes were also characterized [202].

Clearly, there is much to learn from new technologies pertaining to chromosomic studies of birds and the prospect of karyotypes without chromosome preparations is one likely future for these studies. As structure meets function in these investigations, it seems likely that we will gain a deeper understanding of genome evolution in general through examination of this highly fascinating phylogenetic Class. 

## Figures and Tables

**Table 1:** Avian macrochromosome painting data summary.

Order	No. Species Examined	2*n*	Fusions	Fissions	References
Struthioniformes	1	80	No	No	[78]
Rheiformes	2	80	No	No	[78,79]
Casuariiformes	2	80 to 92	No	No	[49,78]
Tinamiformes	1	80	No	No	[78]
Galliformes	15	66 to 82	Yes	Yes	[79,80,81,82]
Anseriformes	8	80 to 98	Yes	No	[76,79,83,84,85]
Apodiformes	3	78 to 80	Yes	No	[86]
Cuculiformes	2	76 to 90	Yes	Yes	[87]
Columbiformes	11	76 to 86	Yes	Yes	[69,79,88,89]
Gruiformes	5	78 to 92	Yes	Yes	[76,90,91]
Charadriiformes	6	42 to 92	Yes	Yes	[32,92,93,94,95,96]
Eurypygiformes	1	80	Yes	Yes	[97]
Ciconiiformes	2	56 to 72	Yes	No	[98]
Pelecaniformes	6	62 to 74	Yes	Yes	[99,100]
Opisthoconiformes	1	80	Yes	Yes	[101]
Accipitriformes	15	58 to 80	Yes	Yes	[73,102,103,104,105,106,107,108,109,110]
Strigiformes	3	76 to 80	Yes	Yes	[79,92,111]
Trogoniformes	1	82	Yes	Yes	[112]
Piciformes	2	112	Yes	Yes	[113]
Falconiformes	3	40 to 52	Yes	Yes	[77,114]
Psittaciformes	10	48 to 72	Yes	Yes	[115,116,117,118,119,120]
Passeriformes	26	60 to 82	Yes	Yes	[68,76,79,88,121,122,123,124,125,126,127,128,129]
Total	126				

**Table 2:** List of avian species that exhibit a fusion on chromosome 4 as revealed by chromosome painting with chicken macrochromosomal paints.

Order	Common Name	Species Name	References
Anseriformes	Greylag goose	*Anser anser*	[79]
Galliformes	Chinese bamboo-partridge	*Bambusicola thoracica*	[81]
Galliformes	Chinese quail	*Coturnix chinensis*	[81]
Galliformes	Common peafowl	*Pavo cristatus*	[81]
Galliformes	Chicken	*Gallus gallus*	[88]
Galliformes	Japanese quail	*Coturnix japonica*	[79,81]
Columbiformes	African collared dove	*Streptopelia roseogrisea*	[79]

**Table 3:** List of avian orders and number of species tested using two selected BACs per reference (chicken) microchromosome.

Order	No. Species Examined	2*n*	Fusions	References
Struthioniformes	1	80	No	[64]
Galliformes	6	78 to 80	No	[64]
Anseriformes	1	80	No	[64]
Caprimulgiformes	1	74	Yes	[140]
Otidiformes	1	76	No	[64]
Cuculiformes	1	74	Yes	[63]
Columbiformes	6	76 to 86	No	[64,89]
Gruiformes	1	92	No	[91]
Charadriiformes	4	78 to 96	Yes	[64,141]
Suliformes	1	74	Yes	[140]
Accipitriformes	1	66	Yes	[110]
Strigiformes	1	80	No	[64]
Trogoniformes	1	82	No	[140]
Piciformes	2	112	No	[140]
Falconiformes	3	50 to 52	Yes	[114,138]
Psittaciformes	4	48 to 72	Yes	[64,119,138]
Passeriformes	7	60 to 80	Yes	[64,142,143,144,145]
Total	42			

## Data Availability

All data are contained within the manuscript and Supplementary Materials.

## Supplementary Materials

The following supporting information can be downloaded at: https://www.mdpi.com/article/10.3390/cells13040310/s1, Table S1: Avian macrochromosome painting data summary per species; Table S2: List of avian species tested using two selected BACs per reference (chicken) microchromosome.
